# Exploring Face Perception Efficiency in Patients with Lacunar Stroke: A Study with Familiar and Unfamiliar Face Recognition

**DOI:** 10.3390/brainsci15101072

**Published:** 2025-09-30

**Authors:** Chi-Yu Lin, Mary Wen-Reng Ho, Sarina Hui-Lin Chien

**Affiliations:** 1 Department of Neurology, Yumin Hospital, Nantou 452, Taiwan; 908699@yumin.com.tw; 2 Graduate Institute of Biomedical Sciences, China Medical University, Taichung 404, Taiwan; 3 Center for Neuroscience and Brain Disease, China Medical University, Taichung 404, Taiwan

**Keywords:** lacunar stroke, face perception, MMSE, mRS, morphing face paradigm

## Abstract

**Background/Objectives**: Stroke is a major cause of disability worldwide, with ischemic stroke being the most common type. This study investigated face perception in patients with lacunar strokes, specifically examining the ability to distinguish and recognize familiar and unfamiliar faces. **Methods**: We tested 52 patients with lacunar stroke (mean age = 65.97 ± 9.96) and 28 age-matched healthy controls (HC) (mean age = 66.24 ± 10.15). The participants received three face perception tasks: Name that Celebrity, Identity Sorting Task, and Face & Object Solitaire, and were also given the MMSE and mRS clinical assessments. **Results:** For the Name that Celebrity task, the stroke group had a lower efficiency score than the control group (i.e., they needed 2–3 extra slides of cues to recognize famous persons). For the Face Identity Sorting task, both groups were more accurate when sorting familiar faces; however, the stroke group performed significantly worse than the healthy group when sorting unfamiliar faces. For the Face/Object Solitaire task, the control group performed better than the stroke group on the face solitaire, but there were no differences in the object solitaire condition. **Conclusions:** Our findings suggest that despite having a normal mean MMSE score (HC: 28.22, Stroke: 27.96), patients with lacunar stroke had difficulties recognizing famous faces and discriminating among unfamiliar faces. This may reveal an overlooked deficit in face perception, highlighting the importance of future interventions that specifically focus on face recognition skills to enhance patients’ daily social interactions and the overall effectiveness of post-stroke rehabilitation programs.

## 1. Introduction

One central goal in neuroscience research is to understand how the brain develops specific cognitive functions. Face perception is of particular interest because faces are fundamental for social interaction and communication. Converging evidence from neuropsychological case reports and brain imaging studies suggests that face processing involves an anatomically and functionally specialized neural circuit located in the occipital and temporal lobes [1,2,3,4,5]. Despite limited visual acuity at birth [6,7], newborns exhibit spontaneous looking preferences towards face or face-like stimuli within days of life [8,9,10,11]. Behavioral and eye-tracking studies further suggest an early cognitive specialization toward realistic face perception [12,13,14]. This early sensitivity is thought to be supported by a subcortical pathway that biases newborns to orient toward faces, thereby providing critical input for the emergence of cortical specialization [15]. Consistent with this account, brain imaging studies on very young infants revealed that infants as young as 2 to 3 months old show evidence of early cortical specialization for processing faces [16,17,18]. Importantly, subcortical structures of the visual system continue to play a role in face processing beyond infancy and are involved in multiple aspects of face perception, from low-level image properties to high-level representations of identity [19]. These findings indicate that cortical face networks develop on the foundation of, and are complemented by, an evolutionarily conserved subcortical system.

Recognizing our caregivers, friends, and family members is crucial for survival, whether as infants, children, or adults [11,20]. The importance of familiarity in face processing has been well established. The early Bruce and Young’s (1986) [21] face recognition model emphasized distinct routes and detailed processes for recognizing familiar and unfamiliar faces. Since then, many studies have demonstrated that recognizing familiar faces is much more accurate and robust than recognizing unfamiliar faces. This is observed in standard face memory tests as well as face matching tests [22,23,24,25,26,27]. Familiarity with faces greatly enhances recognition accuracy, especially in degraded viewing conditions. Research indicates that individuals have difficulty recognizing unfamiliar faces in low-quality images; however, familiar faces can be identified even under the same degraded conditions [28,29].

Stroke is a leading cause of disability worldwide, with an estimated 12.2 million incident cases and 101 million prevalent cases in 2019, resulting in 143 million disability-adjusted life years (DALYs) lost and 6.55 million deaths [30]. It is also the main cause of serious permanent disability and the second leading cause of dementia [31]. Lacunar stroke is a small non-cortical infarct (2–20 mm in diameter) caused by occlusion of a single penetrating branch of a large cerebral artery [32,33]. These branches come from the large arteries of the circle of Willis, the middle cerebral artery (MCA), or the basilar artery. Most lacunar strokes occur in the basal ganglion, subcortical white matter, and pons [34]. According to the Taiwan Stroke Registry, the percentage of disability in patients after acute ischemic stroke is 61.2% within the first month, 55.58% in three months, and 51.72% in six months [35].

The aftermath of a stroke is the leading cause of disability in adults. When evaluating the degree of disability for patients after acute ischemic stroke, physicians in Taiwan often put more emphasis on motor functions and physical disability [36]. However, mild cognitive impairment or very subtle perceptual changes may have been overlooked in stroke patients without marked physical disability. Since lacunar strokes often preserve gross motor function, it is crucial to assess facial recognition abilities in this group, as deficits in face recognition can significantly affect patients’ daily activities, social interactions, and participation in rehabilitation. Systematic evaluation of face recognition can therefore provide a more comprehensive understanding of post-stroke cognitive outcomes and aid in the development of targeted interventions to enhance recovery.

Despite increasing research and rehabilitation concerns on stroke-related deficits like paralysis, aphasia, cognitive impairment [37], and sexuality [38], currently, there is limited information on face processing deficits in patients with acute ischemic stroke. To date, only a handful of studies have explored face and emotion recognition in patients with lacunar stroke [39,40,41]. Using the morphing face technique, Rösler et al. [39] reported that patients with a single infarction in the territory of the right middle cerebral artery (MCA) have more difficulties in recognizing familiar faces than healthy controls and patients with left hemisphere infarctions. They recruited 15 patients with left MCA infarction, 16 with right MCA infarction, with the same Barthel scale (The Barthel scale is an ordinal scale used to measure performance in activities of daily living (ADL). Each performance item is rated on this scale with a given number of points assigned to each level or ranking. From 0 to 100, a higher number is associated with a better performance in ADL.), and 21 age- and sex-matched healthy adults, all screened for moderate or severe dementia via the MMSE. Participants named individuals in two sets of morphing pictures of thirty frames: one transitioning from Jil Sander (a German actress) to Princess Diana (British royal figure) and the other from Michael Douglas (an American actor) to Helmut Kohl (Chancellor of Germany from 1982 to 1990). Healthy participants identified Diana in 25 frames and Kohl in 24. Patients with left-sided stroke recognized both figures slightly later. In contrast, those with right-sided stroke took significantly longer to recognize Kohl and showed more identification failures, highlighting the right hemisphere’s role in familiar face perception.

### Goal and Motivation of the Present Study

The assessment of post-stroke disability primarily focuses on motor impairments and physical limitations. However, mild cognitive and social impairments, such as face recognition, may have been overlooked in stroke patients who do not exhibit significant physical disability. The successful identification of different individuals (i.e., between-person face recognition) or recognizing snapshot photos of the same individuals (i.e., within-person face recognition) is essential for daily social interactions and communications. Despite the importance of these abilities, very few studies have examined face perception in individuals with lacunar stroke.

To address this gap, we investigated familiar and unfamiliar face processing in patients with lacunar stroke and age- and MMSE-matched healthy participants. Specifically, we used three complementary face perception/recognition tasks focusing on three essential aspects: memory for familiar faces, within-person face recognition, and discriminability for unfamiliar faces/objects. The first task was “Name that Celebrity,” which involved a famous face morphing test similar to Rösler et al. (1997) paradigm [39]. This task focused on between-person face recognition and assessed how effectively participants could identify familiar faces as the images transitioned from one person to another. The second task is “Face Identity Sorting,” which involves sorting familiar and unfamiliar face identities. In this task, we tested participants’ within-person recognition abilities to determine how well they could distinguish between individuals and recognize the same person across different images [24,25,26]. The third task was “Face and Object Solitaire,” which required participants to place morphed images of faces or objects in the correct order. This task assessed perceptual discriminability for unfamiliar faces and unfamiliar non-face objects (serving as a control condition).

For the remaining sections of this paper, we illustrate the characteristics of our participants and each of the three tasks in the Materials and Methods (Section 2), followed by the Results (Section 3), which report the clinical assessments and the group mean performances for each task. Additionally, we also investigated the patterns of correlations among age, assessment results, and task performances within both stroke and healthy groups to reveal the potential impact of stroke on these processes. In Discussion (Section 4), we summarize the key noteworthy findings based on each task and their implications for familiar and unfamiliar face perceptions in patients with lacunar stroke, as well as the potential roles of the basal ganglia in face processing. We conclude the Discussion by proposing that face recognition tasks could be a valuable tool for detecting subtle declines in social and cognitive functions in patients with lacunar stroke.

## 2. Materials and Methods

### 2.1. Participants

52 patients with lacunar stroke participated in the study (19 women, mean age = 65.97 ± 9.96). Among these, 36 patients with lacunar stroke did not have diabetes (mean age = 66.47 ± 10.42), while 16 patients had diabetes (mean age = 64.86 ± 9.06), as determined by clinical diagnosis (HbA1C > 6.5%). Because diabetes is associated with dementia, we initially aimed to recruit three groups (stroke patients with and without diabetes, and healthy controls). However, too few stroke patients with diabetes met our criteria, so we analyzed all stroke patients as one group to compare with age-matched healthy controls. All participants in the Stroke group were recruited within the first six months of their first inception. Twenty-eight healthy, age-matched participants (17 women, mean age = 66.24 ± 10.95) were included as the healthy control (HC) group. All participants were recruited from the Neurology outpatient department at Yumin Hospital, Taiwan. Written informed consent was obtained from participants prior to the experiment. The experimental protocols were approved by the Institutional Review Board Committee A of Changhua Christian Hospital, Taiwan (SDF/CCH IRB No.: 181102). To allow reasonable comparisons between stroke patients and healthy controls, all participants completed assessments using the modified Rankin Scale (mRS), Mini-Mental State Examination (MMSE), a visual acuity test, and three face perception tasks. Additionally, all participants filled out a customized evaluation form that included questions about their medical history, location of the stroke (i.e., basal ganglia (L/R/Bilateral), periventricular (L/R/Bilateral), pons, or medulla (L/R/Bilateral)), diabetes, hypertension, and hyperlipidemia. One additional healthy individual was tested but excluded from the analyses because the participant was unable to complete the experiment. Table 1 summarizes the group characteristics, the clinical assessments of the stroke participants.

### 2.2. Stimuli, Apparatus, and Procedures

Participants were tested individually in a quiet, well-lit room during a single clinic visit, where they completed both the clinical assessments and three face perception tasks. These tasks were selected to suit a clinical setting. Each one is brief, effective in targeting specific aspects of face perception, and designed to be engaging and enjoyable for our participants. The first task, “Name that Celebrity,” was administered using a PowerPoint slideshow on a MacBook Air. The second, “Face Identity Sorting,” and the third, “Face & Object Solitaire,” tasks were presented using printed and laminated cards, allowing for flexible and interactive testing. This format helped maintain the participants’ interest and attention while also ensuring that the tasks could be administered efficiently within a limited time frame.

#### 2.2.1. Assessment of mRS, MMSE, and Visual Acuity

All participants underwent the Modified Rankin Scale (mRS) and the MMSE assessments before completing the three face perception tasks. The modified Rankin scale (mRS) is commonly used to evaluate the severity of physical disability or dependence in daily activities for individuals who have experienced a stroke or other neurological diseases. The mRS also has the broadest application in evaluating clinical outcomes for stroke clinical trials [42]. The mRS uses a seven-point scale, covering the full spectrum of functional outcomes from no symptoms (0) to death (6). A trained experimenter determined the patients’ scores with a structured interview. The Mini-Mental State Examination (MMSE) [43] is a 30-point questionnaire commonly used to assess cognitive functions and screen for dementia. The test encompasses several domains of cognitive function, including orientation, registration, attention, calculation, recall, language, repetition, and the ability to follow simple commands. The test takes about 10 min to complete. For the visual acuity test, we used the EyeChart (Dok LLC, Largo, FL, USA) [44]. This pocket vision screen app features a randomized Landolt C Chart to assess the visual acuity of all participants. If a participant’s vision was below the average (20/30), they were encouraged to use corrective eyeglasses or bifocals. We used the ratio as a measure of visual acuity (i.e., 20/20 = 1.0).

#### 2.2.2. Task 1: Name That Celebrity

In this task, two famous headshot photos, one of a celebrity and one of a politician from each gender, were selected as the stimuli (i.e., following the design of Rösler Et Al., 1997 [39]). The color headshot photos were all taken in a frontal view with a natural smile. We used FantaMorph 5 Deluxe (Abrosoft Co. Nebraska, USA) to morph the female faces (Pai Bing-Bing, a well-known Taiwanese singer and actress; and Chen Chu, the former mayor of Kaoshiung city from 2006 to 2018) and the male faces (Lai Ming, a well-known Hong Kong actor and singer; and Ma Ying-Jeou, the former president of Taiwan from 2008 to 2016). Thus, two morph sets, each comprising 19 morphing images with a 5% interval, were created. A total of 54 key corresponding points on the facial features were kept constant for each of the two original faces (i.e., 8 points on each eye, 10 points on the nose, 11 points on the mouth, 4 points on each eyebrow, and 9 points for the contour) (please see [45] for the detailed morphing procedures). The female set contained the original face of Pai (0%, denoted as Pai 100/Chen 0), and 5% (adding 5% of the original Chen’s face to the original Pai’s face), 10%, 15%, 20%, 25%, 30%, 35%, 40%, 45%, 50%, 55%, 60%, 65%, 70%, 75%, 80%, 85%, 90%, 95% morphed images, as well as the original face of Chen (100%, denoted as Pai 0/Chen 100). Likewise, the male set contained the original Lai (0%), the original Ma (100%), and 19 mixed morph faces.

Two additional sets of morphed images, with a 10% interval (a total of 11 slides), were created to serve as practice stimuli. Female participants completed a practice trial using the male stimuli, which consisted of 11 slides (with a 10% interval). Participants viewed 11 slides one at a time during the practice session. For each slide, participants were asked to identify the person depicted, resulting in a total of 11 responses for practice. We recorded the number of the first slide on which the participant identified the target politician and correctly called out their name. Following this, the participants proceeded to the formal trial, which featured the female stimuli and consisted of 21 slides with a 5% morphing interval. Conversely, male participants began with the practice trial of female stimuli (11 slides at a 10% interval) and then proceeded to the formal trial of male stimuli (21 slides at a 5% interval). In this task, participants were shown a series of morphed images that gradually transformed one person into another. For each slide, we confirmed whether participants recognized the face. If a participant recognized the celebrity or politician, they indicated their answer by stating the name, either Pai or Chen for the female stimulus set, or Lai or Ma for the male stimulus set. The specific slide number where they first identified the target celebrity by calling out the name was carefully recorded. If they did not recognize the celebrity, they had the option to respond with “unknown.” This process continued for each slide until the final image was presented, ensuring that participants could engage fully with the task throughout. The duration for completing Task 1 was between 2 and 3 min.

#### 2.2.3. Task 2: Face Identity Sorting

For the familiar condition, 20 pictures of each of the two well-known Taiwanese male celebrities, Hu Gua and Chang Fei (both are best known for hosting various entertainment TV shows in Taiwan and are popular among people of all ages), were downloaded from the internet using Google Images. For the unfamiliar condition, twenty pictures of the two Asian celebrities, John Wu (a Hong Kong film director, producer and screenwriter with several notable action films) and Haruki Murakami (a famous contemporary Japanese writer, whose books have been translated into over 50 languages), whose faces were unknown to most Taiwanese people were downloaded via Google Image from internet. The selected pictures were mostly frontal face photos with different expressions, hairstyles, viewing angles, and lighting conditions. All photos were first converted to grayscale to reduce differences in skin tone and background using PhotoImpact 10 (Ulead Systems, Taipei, Taiwan). Each image measured 108 pixels in width and 142 pixels in height. The face stimuli were then printed on cardboard and laminated. Each laminated face card measured 4 cm in width by 5.1 cm in height.

Participants performed the face identity sorting task at the clinic. Each participant received two conditions, each containing one formal trial with 40 photos to sort by perceived identity. All participants first sorted the unfamiliar condition, then the familiar one. This fixed test order was designed to strategically reduce the chance that participants may spontaneously realize there were only two identities (for more details on the design, see Ali & Chien [46]). They received 40 photos of unfamiliar celebrities and were told, “Please look at these photos and sort them into piles based on identity. Put the same identity into the same pile. You can make as many or as few piles as you like.” There were no time limits or restrictions on the number of groupings that the participants could create. After finishing, the experimenter took a photo of the sorted photos facing up, then turned the photos face down to take a picture from the back. The same procedure was repeated for the second set. After finishing the task, participants were asked if they recognized any of the individuals in the photos. If they said yes, we inquired about that person’s name. If they were unfamiliar, they simply stated they did not recognize anyone. In the Familiar condition, participants who did not recognize either of the two Taiwanese famous celebrities were excluded from further analysis. The typical duration for completing Task 2 was 7 to 10 min, with about 2–3 min for the Familiar condition and 5–8 min for the Unfamiliar condition.

#### 2.2.4. Task 3: Face and Object Solitaire

The solitaire task consists of a face block and a non-face object block. All participants completed a practice trial to familiarize themselves with the object and face blocks before the formal test trial.

##### Morphing Face Solitaire Task

We used one female Asian face selected from the Taiwanese Facial Expression Image Database, TFEID [47], and one female Caucasian face selected from the NimStim Face Stimulus Set [48]. The face photos were in a frontal view with a neutral expression. All faces were first converted to gray-scale to minimize the difference in skin tone. The face images were then oval-cropped to remove the external non-facial features such as the hair, the ears, and clothing via Windows 7 Paint and PhotoImpact 10 (Ulead System, Taipei). FantaMorph 5 Deluxe was used to mix these two original faces (one Asian face and one Caucasian face) and created four morphing images with 20% interval on a linear continuum (i.e., A20/C80, A40/C60, A60/C40, and A80/C20) (i.e., the face stimuli were taken from a subset of [49,50]). Figure 3A illustrates the original face stimuli and the four morphing face stimuli. All of the face stimuli were printed out on cardboard paper and laminated. Each laminated face card was 7.5 cm (width) by 9.5 cm (height).

##### Morphing Teapot Solitaire Task

The photos of two different teapots (denoted as teapot A and teapot B) with similar appearances, orientations, and sizes were downloaded from the internet. The two teapots were first converted to gray-scale images. Likewise, we used FantaMorph 5 Deluxe to create four morphing images with a 20% interval on a linear continuum (i.e., A80/B20, A60/B40, A40/B60, and A20/B80). The teapot images were taken from a stimulus subset of [51]. All the morphed stimuli were printed on cardboard paper and laminated. Each object card was 8.5 cm (width) by 11 cm (height).

Before the test, two practice trials with different faces and objects were given to acquaint the participants with the task. At the beginning of the test trial, the experimenter placed the two original face cards (Asian 100, Caucasian 100) or the two original teapot cards (teapot A 100, teapot B 100) at the very end of the row as the anchoring reference cards, and left enough space in the middle for participants to place the four morphed face or teapot cards. The locations of the two anchoring cards were counterbalanced between participants. In each trial, participants were given four randomly pre-shuffled morphed face cards (or pre-shuffled morphed teapot cards) and were instructed to place these cards based on perceived similarity, transitioning gradually from one face to another (or one teapot to another). After the participant completed the task, the experimenter took a photo and recorded the positions of the cards as placed by the participant on a customized scoring sheet. The duration for completing Task 3 was between 1.5 and 2 min.

## 3. Results

Table 1 summarizes the group characteristics, the clinical assessments, and the locations of lacunar infarcts of the stroke participants. To make a more reasonable comparison between the Stroke and HC groups, all participants underwent an MMSE assessment before the three face perception tasks. There were no significant group differences for MMSE score, age, years of education, or visual acuity (see Table 1). Table 2 summarizes the group mean performance of the three main tasks.

### 3.1. Performance for Task 1: Name That Celebrity

First, for each participant, we calculated an Efficiency score as a performance measure to represent how effectively they identified the target celebrity. The Efficiency score is defined as $\frac{\left(21-slide\#\right)}{21}$, in which 21 is the total number of slides and slide # is the slide that the participant correctly identified the target. The Efficiency score ranges from 0 to 1; a score of 0.48 means the participant correctly identified the target on slide #11, which is the midpoint corresponding to the 50% morphing level. A score above 0.48 indicates the participant identified the target celebrity before slide 11, while a score below 0.48 suggests they needed more slides to identify. An Efficiency score of 0 denoted that the participants could not recognize the target until the last slide (100% original photo of the celebrity) or failed to recognize the target celebrity.

**Figure 1:**
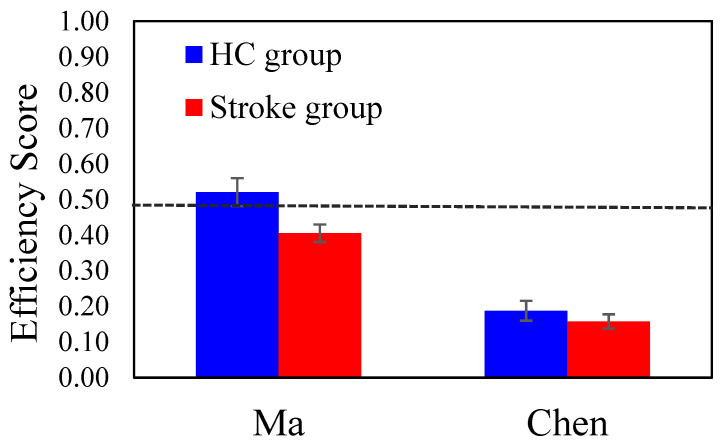
The main results of Task 1: Name That Celebrity, showing the group efficiency score ($\frac{\left(21-slide\#\right)}{21}$), where 0.48 corresponds to the 50% morphing level. The blue and red colors represent the HC and the Stroke groups, respectively. The error bars represent the standard errors (*SE*) of the means.

A 2-way ANOVA with *Group* (HC Vs. Stroke) and *Stimuli Gender* as the between-subject factors was conducted with the Efficiency score. The *Group* main effect was significant (*F* (1, 76) = 5.355, *p* = 0.024, η_p_^2^ = 0.066); the mean efficiency score of the HC group (*M* = 0.376, *SE* = 0.033) was higher than that of the stroke group (*M* = 0.282, *SE* = 0.024). The main effect of *Stimuli Gender* was significant (*F* (1, 76) = 51.223, *p* < 0.001, η_p_^2^ = 0.403); the mean efficiency score for the male celebrity (Ma) (*M* = 0.475, *SE* = 0.030) was higher than that of the female celebrity (Chen) (*M* = 0.183, *SE* = 0.027). The *Group × Stimuli Gender* interaction was not significant (*p* = 0.282). Figure 1 shows the group mean efficiency scores for the male celebrity (Ma) and the female celebrity (Chen) conditions. Overall, patients in the Stroke group exhibited a significantly lower efficiency score, meaning that they required more cues to recognize the target celebrity compared to the healthy controls. Furthermore, within both groups, the recognition of Ma (i.e., the former president of Taiwan) yielded a higher efficiency score than that for Chen.

### 3.2. Performance for Task 2: Face Identity Sorting

In this task, we focused on two performance indices commonly used in the card sorting task [24,26]: *the number of sorted piles* (indicating the robustness of within-person recognition) and *the number of misidentification errors* (indicating whether the participants made an error in between-person recognition). Three Stroke patients and two control participants were excluded from the final analysis due to incomplete data, as they either did not complete the familiar condition or the unfamiliar condition.

#### 3.2.1. The Number of Sorted Piles

**Figure 2:**
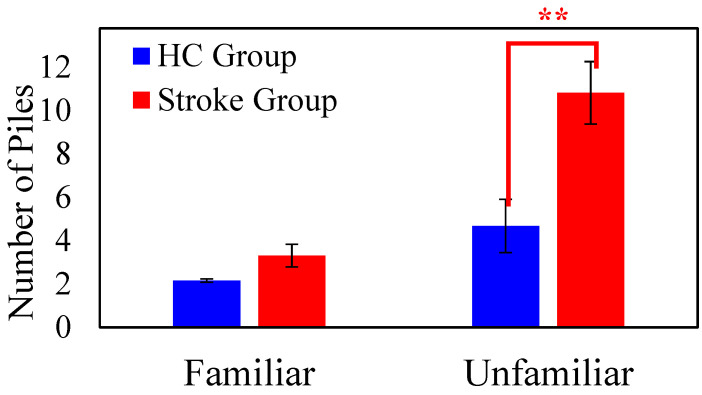
The main results of the Task 2: “Face Identity Sorting” showing the group mean numbers of sorted piles for the Familiar and Unfamiliar face conditions. The blue and red colors represent the HC and the Stroke groups, respectively. The error bars represent the standard errors (*SE*) of the means. (** *p* ≤ 0.01).

We conducted a 2-way mixed ANOVA with *Group* (HC vs. Stroke) as the between-subject factor and *Familiarity* as the within-subject factor. The *Group* main effect was significant (*F* (1, 71) = 9.541, *p* = 0.003, η_p_^2^ = 0.118); the mean number of piles of the HC group (*M* = 3.077, *SE* = 1.074) was significantly lower than that of the Stroke group (*M* = 7.213, *SE* = 0.799). The main effect of *Familiarity* was significant (*F* (1, 71) = 23.019, *p* < 0.001, η_p_^2^ = 0.245); the mean number of piles of the Familiar condition (*M* = 2.726, *SE* = 0.360) was lower than that of the Unfamiliar condition (*M* = 7.564, *SE* = 1.129). Importantly, the *Group × Familiarity* interaction effect was significant (*F* (1, 71) = 8.803, *p* = 0.004, η_p_^2^ = 0.110). In the Familiar condition, the mean number of sorted piles of the stroke group (*M* = 3.298, *SE* = 0.429) was slightly greater than the HC group (*M* = 2.154, *SE* = 0.577), but the difference was not significant (*p* = 0.117). The group difference was augmented in the Unfamiliar condition; the mean number of sorted piles of the stroke group (*M* = 11.128, *SE* = 1.348) was significantly greater than that of the HC group (*M* = 4.000, *SE* = 1.812, *p =* 0.004). Figure 2 shows the group mean performances for the Familiar and the Unfamiliar face conditions. Both groups sorted fewer than three piles in the familiar condition. However, for the unfamiliar condition, the Stroke group sorted significantly more piles than the Healthy Control group. This indicates that recognizing the unfamiliar condition was more challenging for the Stroke group.

In addition to comparing the group means, we also examined the distribution of individuals’ performance with the Chi-square test of independence. According to the participants’ performance, we divided them into two categories: ‘two-pile’ group and ‘more-than-two-pile’ group. In the Familiar face condition, 22 healthy participants sorted two piles, and 4 sorted more than two piles; 35 stroke patients sorted two piles, and 13 sorted more than two piles. The χ^2^ test of independence was not significant, χ^2^ (1, 74) = 1.304, *p* = 0.253, meaning that both groups had a similar distribution. In the Unfamiliar face condition, 16 healthy participants sorted two piles, and 10 sorted more than two piles; 9 stroke patients sorted two piles, and 42 sorted more than two piles. The χ^2^ test of independence was highly significant, χ^2^ (1, 77) = 15.130, *p* < 0.001. The analysis of the individual’s distribution aligned with the ANOVA results; the stroke group tended to sort into more than two piles more often than the healthy controls, especially in the more difficult Unfamiliar face condition.

#### 3.2.2. The Number of Misidentification Errors

We conducted a 2-way mixed ANOVA on misidentification errors with *Group* (HC vs. Stroke) as the between-subject factor and *Familiarity* as the within-subject factor. The *Group* main effect was not significant (*p* = 0.687); the mean number of misidentification errors of the HC group (*M* = 0.346, *SE* = 0.094) was not different from that of the Stroke group (*M* = 0.394, *SE* = 0.070). The main effect of *Familiarity* was not significant (*p =* 0.309); the mean number of errors in the Familiar condition (*M* = 0.318, *SE* = 0.068) was not significantly different from that of the Unfamiliar condition (*M* = 0.422, *SE* = 0.086). The *Group × Familiarity* interaction effect was not significant (*p* = 0.221). In short, both groups rarely made misidentification errors in either condition (the means were less than 1 in both groups).

### 3.3. Performance for Task 3: Face & Object Solitaire

We conducted a 3-way mixed ANOVA on accuracy with *Group* (HC vs. Stroke) as the between-subject factor, *Card Type* (Face vs. Object) and *Position* (1, 2, 3, and 4) as the within-subject factors. The *Group* main effect was not significant (*p* = 0.143); the mean accuracy of the HC group (*M* = 0.804, *SE* = 0.049) was slightly higher than that of the stroke group (*M* = 0.714, *SE* = 0.036), but the difference was not significant. The main effect of *Card Type* was not significant (*p* = 0.870); the mean accuracy of the Face card (*M* = 0.754, *SE* = 0.041) was similar to the Teapot card (*M* = 0.763, *SE* = 0.039).

**Figure 3:**
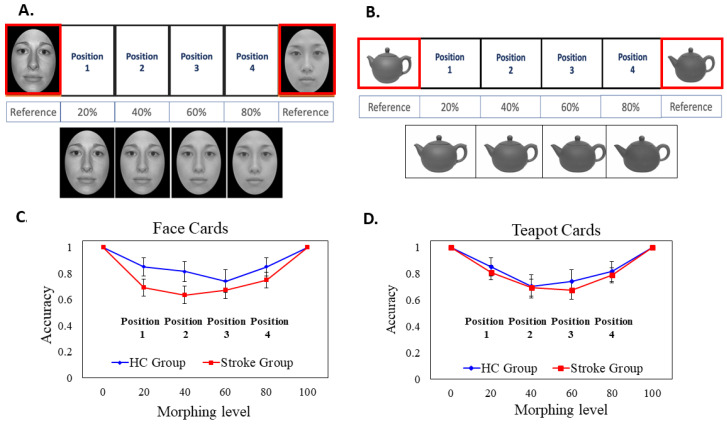
The stimuli and main results of Task 3: Face/Object Solitaire. Panel (**A**,**B**): Illustrations of the “Face Solitaire” and “Teapot Solitaire” tasks. The orange box highlights the two original face/teapot reference cards (serving as the anchors). Panel (**C**,**D**): The group mean accuracies for the face and teapot solitaire at four different positions. The blue and red colors represent the HC and the Stroke groups, respectively. Please note that the data at the two endpoints (0 and 100) are hypothetical and not actual data. The error bars represent the standard errors (*SE*) of the means.

The main effect of *Position* was significant (*F* (3, 76) = 5.953, *p* = 0.001, η_p_^2^ = 0.190). Post hoc pairwise comparisons (with an adjusted α level = 0.05/6 = 0.008) revealed that the mean accuracy of the Position 1 (*M* = 0.804, *SE* = 0.036) was significantly higher than that of Position 2 (*M* = 0.716, *SE* = 0.039, *p* = 0.001), and marginally higher than Position 3 (*M* = 0.712, *SE* = 0.040, *p* = 0.048). The mean accuracy of Position 4 (*M* = 0.804, *SE* = 0.038) was significantly higher than Position 3 (*p* < 0.001) and marginally higher than Position 2 (*p* = 0.048). None of the two-way or three-way interaction effects was significant. Although the interaction between group and position was not significant, exploratory *t*-tests were conducted to examine the potential position-specific group differences. Descriptively, Positions 2 and 3 appeared more difficult than Positions 1 and 4, as the former two positions were further away from the anchoring faces or teapots. For the Face Solitaire, Position 2 was a Caucasian-Asian mixed face with more than 50% Caucasian component. Due to the potential other-race effect, we considered Position 2 to be the most challenging among the four. As we were interested in the more difficult Position 2, an independent *t*-test revealed that the accuracy of Position 2 of Face cards of the Stroke patients (M = 0.635, SE = 0.067) was significantly lower than that of the HC group (M = 0.821, SE = 0.074), *t*(76) = −1.751, *p* = 0.042. However, there was no group difference for Position 2 of the Object card. Figure 3, Panels C and D, illustrates the group mean accuracies of the face and the teapot solitaire, showing the four different positions (note that the data at the two endpoints (0 and 100) are hypothetical and not actual data).

### 3.4. Correlations Among Age, the Assessments, and the Task Performances

To further examine the relationships among the participants’ age, clinical assessments (MMSE), and the performance on the three tasks (i.e., the Efficiency score, the number of sorted piles for familiar and unfamiliar conditions, and the accuracy for face and object solitaire), we conducted Pearson’s correlations for each group separately. Table 3 summarizes the correlation strengths and *p*-values for the Stroke and HC groups. In the HC group, age negatively correlated with efficiency score (*r* = −0.438, *p* = 0.020), and the accuracy of face solitaire task (*r* = −0.420, *p* = 0.021), indicating that the older healthy participants needed more cue to recognize the target celebrity and tended to make more errors in the face solitaire. We also observed a significantly positive correlation between MMSE and the face solitaire accuracy (*r* = 0.484, *p* = 0.009) and a marginal correlation with the object solitaire task (*r* = 0.364, *p* = 0.057). Furthermore, the accuracy of face solitaire was strongly correlated with performance in identity sorting of the unfamiliar condition (*r* = −0.467, *p* = 0.012), meaning that those who did better with face solitaire also tended to sort fewer piles in the unfamiliar face card sorting task.

In the Stroke group, age negatively correlated with the accuracy of the face solitaire task (*r* = −0.421, *p* = 0.002), positively correlated with familiar face sorting task (*r* = 0.325, *p* = 0.024), and unfamiliar face sorting task (*r* = 0.464, *p* = 0.001), indicating that older patients in the stroke group tended to perform worse with face perception related tasks. Importantly, the individual’s MMSE did not correlate with the three tasks, implying that while a patient might have a normal MMSE score; they could still have difficulty in specific areas such as face processing. Furthermore, the efficiency score positively correlated with the accuracy of face solitaire task (*r* = 0.324, *p* = 0.019), but not with the object solitaire. The number of piles in the unfamiliar face sorting negatively correlated with the efficiency score (*r* = −0.308, *p* = 0.028) and the accuracy of face solitaire task (*r* = −0.243, *p* = 0.086), indicating that the patient who were faster to identify the target celebrity, or did better in the face solitaire task also tended to show better within-person face recognition (i.e., sorting fewer piles).

## 4. Discussion

This study is among the first to explore familiar and unfamiliar face perception in lacunar stroke patients within three years post-stroke and their age-matched healthy controls. We evaluated each participant’s face processing ability through three specific face perception tasks and assessed their general cognitive function using a conventional neuropsychological assessment (MMSE). We observed several noteworthy findings. First, for between-person face recognition of well-known individuals, compared to healthy controls, the stroke patients required more cues to identify the target celebrity, but they were not completely unable to recognize familiar celebrities. Second, for within-person face recognition, both groups were more accurate in sorting photos of familiar individuals than in sorting photos of unfamiliar individuals, indicating the robustness effect of familiarity. However, the stroke group sorted significantly more piles than the healthy group in the unfamiliar face condition, suggesting that unfamiliar person recognition is more fragile for patients with lacunar stroke. Third, we found that when participants were asked to arrange the morph cards in the correct order, the healthy group performed better than stroke patients in one of the more challenging positions in face solitaire. However, both groups demonstrated similar performance for the object solitaire, suggesting that the impairment is specific to face stimuli. Moreover, it is important to note that the MMSE scores for both groups showed no difference, suggesting that their cognitive performance in standard clinical assessments was comparable. These findings indicate that stroke patients, especially those who are older, experienced notable challenges in face recognition tasks when compared to their age-matched healthy counterparts. This suggests that even when standard cognitive function assessments, such as the MMSE, appear normal, more thorough evaluations focusing on face perception/recognition may reveal hidden deficits.

### 4.1. Familiar Face Recognition Is Preserved but Less Efficient

The ‘Name that Celebrity’ task aimed to assess the mental representations of well-known individuals. An efficiency score was calculated as a performance index to reflect the effectiveness of identifying the target face from a series of progressively morphing face images. Detecting subtle changes in morphing stimuli necessitates a combination of featural (i.e., the shape of the mouth), holistic (integrating features together), and second-order configural (the distances between features) processing [49,50,51]. We found that the Stroke group needed a greater physical difference to produce a detectable change; in other words, they needed more cues to ‘recognize’ the celebrity. Our findings are consistent with Rösler’s (1997) study [39], which also utilized a similar morphing face paradigm (Female stimuli: Jil Sander morphed into Lady Diana; Male stimuli: Michael Douglas transformed into Helmut Kohl). Each participant viewed two sets of 30 images that gradually transitioned from one person to another on a video screen. They found that patients with a single infarction in the territory of the right middle cerebral artery (MCA) had more difficulties (i.e., required more frames) in recognizing familiar faces than healthy controls and patients with left hemisphere infarctions. Nevertheless, it is worth noting that our stroke patients were not completely unable to recognize familiar celebrities; rather, they required additional cues to do so. This suggests a partial impairment in accessing stored identity representations rather than a complete failure of recognition.

In the ‘Face Identity Sorting’ task, which was adapted from previous studies [24,26], we incorporated two conditions: the Familiar condition, featuring well-known Taiwanese male celebrities Hu Gua and Chang Fei, and the Unfamiliar condition, featuring faces unfamiliar to Taiwanese participants, specifically Japanese writer Murakami Haruki and Hong Kong director John Wu. This design was structured as a within-subjects experiment [46]. We found that both groups could accurately sort the familiar individuals’ photos into two piles. Although the Stroke patients produced slightly more piles for the familiar condition compared to the HC group, the difference did not reach statistical significance, indicating that recognition of familiar faces was relatively preserved, even in the case of lacunar stroke. Our results support the idea that familiar face perception is highly robust, as shown by previous studies using a similar card sorting method [24,26], despite significant differences in experimental design and the participants’ ages. While Jenkins (2011) tested young participants, we recruited stroke patients aged between 45 and 86 years (with a mean age of 66) and age-matched healthy adults. Our study demonstrated the familiarity effect in patients with lacunar stroke and their age-matched healthy controls, similar to that previously reported in young adults and children [24,52,53,54,55].

### 4.2. Unfamiliar Face Recognition Is Fragile in Lacunar Stroke

Importantly, in the Unfamiliar Face condition, the stroke group was found to sort significantly more piles than the healthy group, indicating the impairment was more pronounced in the unfamiliar condition. In addition to the averaged group performances, a striking difference was also observed when examining individuals’ performance: 11 participants in the stroke group sorted more than 20 piles, with a maximum of 38 piles sorted, whereas only one participant in the healthy group sorted more than 20 piles in the Unfamiliar condition. This also indicates that patients had greater difficulty forming or maintaining stable identity representations when the faces lacked pre-existing familiarity. We believe these findings reflect the subtler gradations in face processing deficits—familiarity offers some compensatory advantage, but patients still show signs of weakened identity processing. We believe that subcortical stroke lesions may hinder the low-level aspects of face processing, such as image contrast and viewpoints. This disruption can complicate the recognition of unfamiliar faces, particularly under varying conditions of illumination, poses, and expressions. This aligns with the findings of Gabay et al. [20], suggesting that the pre-striate subcortical areas play a critical role in perceiving low-level image properties and forming representations of face identity.

Additionally, among the 12 participants (11 patients and one healthy control) who sorted more than 20 piles of unfamiliar face cards, we observed a high prevalence of hyperlipidemia comorbidity alongside statin treatment. This finding suggests that elevated blood lipid levels, recognized as risk factors for intracranial atherosclerosis and cardiovascular disease, may subtly impair blood flow to brain regions involved in visual cognitive tasks, such as the structural analysis of new or unfamiliar faces.

### 4.3. Face Discrimination Deficits Are Specific to Facial Stimuli

The ‘Face & Object Solitaire’ task involved a face block with an Asian female face and a Caucasian female face, which were combined to create image cards. Participants sorted these cards according to their similarity. Likewise, two teapots of comparable styles were used to generate morphed teapot image cards for sorting. It is essential to note that this task was designed to investigate group differences in the ability to discriminate unfamiliar faces and objects, and to test whether the impairment is specific to faces. The results showed that the HC group had a slightly higher average accuracy across positions than the stroke group in the face condition. However, this difference was not statistically significant when combining the accuracies for all four positions. When examining location-specific performances, we found that the accuracy of Position 2 for face cards among stroke patients was significantly lower compared to the HC group. Most critically, this specific impairment in processing the more challenging position was not observed in the object condition. In patients with lacunar stroke, the infarct locations are mainly near the basal ganglia, which do not involve the known cortical regions specialized for face processing. However, we observed a reduced perceptual discriminability for unfamiliar faces with subtle changes, but not for unfamiliar objects with subtle changes in stroke patients.

### 4.4. Clinical Assessments May Miss Subtle Face Processing Deficits

When analyzing Pearson’s correlations among participants’ age, cognitive assessment scores (MMSE), and task performance, we observed several noteworthy trends for each group (see Table 3). For the healthy participants, we found an effect of age, in that older participants tended to show lower efficiency scores (i.e., needed more cues) and lower accuracy in the face solitaire task. There was also a strong positive connection between the MMSE score and accuracy in the face solitaire task, which was absent in the Stroke group. Additionally, we noted that better accuracy in face solitaire was associated with sorting fewer piles in the unfamiliar condition, showing a connection between between-person face discrimination and within-person face recognition.

In the Stroke group, we also observed that age significantly impacted performance on face recognition tasks. Older patients tended to perform worse on the face solitaire task as well as both the Familiar Face Sorting and the Unfamiliar Face Sorting task (i.e., tended to make more piles). Interestingly, we found that the MMSE scores did not correlate with performance across these tasks. This suggests that even patients who achieve normal MMSE scores may still have difficulties with perceptual and cognitive tasks related to face processing. We also found correlations among the performances of the three tasks; the efficiency score was positively correlated with the accuracy of the face solitaire task (but not with the object solitaire task) and negatively correlated with the number of piles in the unfamiliar face sorting task, showing a connection between between-person face discrimination and within-person face recognition.

### 4.5. Subcortical Lesions May Disrupt the Face Processing Network

Haxby and Gobbini [4,5] proposed a distinction between the Core System and the Extended System in the context of face recognition. The Core System comprises three bilateral regions within the occipital-temporal extrastriate visual cortex that are specifically involved in viewing faces, as opposed to other visual categories. In contrast, the Extended System encompasses regions outside the extrastriate visual cortex, including the prefrontal cortex, temporal cortex, temporoparietal junction, and posterior cingulate cortex, which collectively contribute to the analysis of person knowledge, facial expressions, and familiarity with individuals. We believe that the cerebral cortex plays a crucial role in face recognition and visual perception. Additionally, the interaction between the basal ganglia and the cortex is significant and may enhance our understanding of how both the Core and Extended Systems function together.

As previously explained, lacunar stroke is caused by small non-cortical infarcts due to the occlusion of a single penetrating branch of a large cerebral artery [32,33]. These branches originate from the large arteries of the circle of Willis, the stem of the middle cerebral artery (MCA), or the basilar artery. Most lacunar strokes occur in the basal ganglion, followed by the subcortical white matter and pons [34]. The etiology is described as lipohyalinosis or microatheroma of the penetrating arteries. The basal ganglion not only regulates motor control but also receives extensive inputs from the cerebral cortex, the substantia nigra, and the lateral amygdala [56]. Damage to the basal ganglia has been associated not only with motor dysfunction but also with emotional incontinence or blunting [57,58] and cognitive impairments in executive function, memory, and attention [59].

In the present study, we found that stroke locations in patients with lacunar strokes were primarily situated near the basal ganglia, unilateral or bilateral. Notably, these lesions did not involve the three core face processing regions within the occipital-temporal visual cortex. However, we did observe a decline in perceptual discriminability for unfamiliar faces, as evidenced by difficulties in both the face solitaire task for between-person recognition of unfamiliar faces and the photo sorting task for unfamiliar within-person face recognition. This suggests that, although the key cortical areas involved in face processing remained intact, damage to the basal ganglia may affect face processing. Lesions in the vicinity of the basal ganglia, coupled with the lack of support from the extended system, could therefore collectively contribute to a reduced perceptual discriminability for unfamiliar faces with subtle differences.

### 4.6. Implications for Rehabilitation Strategies

Last but not least, previous research on rehabilitation for individuals with face recognition difficulties, usually those with acquired prosopagnosia, has examined various strategies, including perceptual training and compensatory techniques. Early studies have summarized different methods and their potential effectiveness in enhancing recognition performance. These reviews highlight both the potential benefits and the limitations of current interventions, emphasizing the need for continued development of evidence-based rehabilitation programs tailored to different patient groups [60,61,62]. Based on this, our findings suggest that rehabilitation strategies may involve targeted face discrimination training and enhancing configural processing, such as personalized face recognition exercises using ambient photos with natural variations in lighting, pose, and expressions. Incorporating these strategies may help stroke patients improve their face recognition skills and enhance their social interactions.

### 4.7. Limitations of the Current Study and Future Directions

The present study had several limitations. First, only one to two trials per task were conducted due to strict time constraints in the clinical setting. While this approach allowed us to capture essential data within practical limits, it may reduce the robustness of performance measurement. Second, the sample sizes were uneven, with nearly twice as many stroke patients as healthy controls. Our initial plan was to recruit three groups: stroke patients with diabetes, stroke patients without diabetes, and healthy controls. However, during recruitment, we found that the number of stroke patients with diabetes meeting our criteria was very limited, making it impossible to gather enough participants for this group. As a result, we excluded the diabetes factor and combined all stroke patients into a single group, then compared their performances with those of age-matched healthy controls. Third, the lesion sites among stroke patients were highly heterogeneous, with 39 patients having major lesions near the basal ganglia, 8 near the periventricular region, and 6 near the brainstem areas. Additional factors such as hyperlipidemia and hypertension may also have influenced outcomes. Given the small number of patients within each lesion subgroup, we were unable to further examine correlations between infarction loci and face recognition performance.

Future directions based on the aforementioned limitations include conducting studies with larger and more balanced sample sizes, implementing multiple trials per task to improve measurement reliability, and systematically investigating how lesion location, comorbidities such as diabetes or hyperlipidemia, and medication use (e.g., statins) interact to influence face perception and memory.

## 5. Conclusions

The present study investigated face perception in patients with lacunar stroke, specifically examining their ability to recognize familiar and unfamiliar faces. Overall, the patients could recognize familiar individuals but needed more cues to identify celebrities’ faces. In identity sorting, both groups were better at sorting images of familiar celebrities; however, stroke patients performed poorly when sorting unfamiliar photos. In the face solitaire task, healthy controls were more accurate than stroke patients in the more difficult face solitaire task; however, they performed equally well in the object solitaire task, suggesting that the difficulty is limited to face stimuli. Lastly, Pearson’s correlations revealed that performance on all three tasks did not correlate with MMSE scores in the stroke group. This indicates that even patients who achieve normal MMSE scores may still experience difficulties with face processing. Our findings underscore the necessity for rehabilitation strategies that could incorporate targeted face discrimination training using ambient photos to enhance within-person face recognition.

## Figures and Tables

**Table 1:** Participants’ demographics and characteristics (Means and SD).

Characteristics	Stroke (*n* = 52)	HC (*n* = 28)	*p*-Value
Age	65.97 ± 9.96	66.24 ± 10.15	0.912
Gender	F: 19 M: 33	F: 18 M: 10	0.025 *
Education ^1^	10.04 ± 3.17 (yrs)	11.21 ± 4.34 (yrs)	0.169
Visual Acuity ^2^	0.72 ± 0.27	0.74 ± 0.22	0.885
MMSE ^3^	27.96 ± 1.55	28.22 ± 1.58	0.482
mRS ^4^	1.19 ± 0.60	0	<0.001 ***
Stroke Location			
Basal Ganglia (L/R/Bilateral)	8 (L), 9 (R), 22 (Bilateral)	N/A	N/A
Periventricular (L/R/Bilateral)	3 (L), 5 (Bilateral)	N/A	N/A
Pons (L/R/Bilateral)	2 (L), 2 (R)	N/A	N/A
Medulla (L/R/Bilateral)	2 (L)	N/A	N/A

^1^ Education: the years of formal education; ^2^ Visual Acuity: the ratio as a measure (i.e., 20/20 = 1.0); ^3^ MMSE: Mini-Mental State Examination. ^4^ mRS: Modified Ranking Scale. * *p* < 0.05, ** *p* ≤ 0.01, *** *p* ≤ 0.001.

**Table 2:** Summary of the participant’s task performance (Means and SE).

Tasks	Stroke (*n* = 52)	HC (*n* = 28)	*p*-Value
Task 1: Name That Star			
Efficiency Score (mean)	0.28 ± 0.024	0.376 ± 0.033	0.024 *
Efficiency Score (male celebrity)	0.41 ± 0.025	0.52 ± 0.04	0.040 *
Efficiency Score (female celebrity)	0.16 ± 0.02	0.20 ± 0.03	0.189
Task 2: It Has to be You			
Number of Piles (familiar)	3.31 ± 0.52	2.15 ± 0.07	0.054
Number of Piles (unfamiliar)	10.82 ± 1.44	4.69 ± 1.18	0.003 **
Misidentification (familiar)	0.40 ± 0.09	0.23 ± 0.08	0.113
Misidentification (unfamiliar)	0.39 ± 0.10	0.46 ± 0.14	0.345
Task 3: Face/Object Solitaire			
Accuracy for Face	0.69 ± 0.05	0.82 ± 0.05	0.054
Accuracy for Object	0.74 ± 0.05	0.79 ± 0.05	0.281

* *p* < 0.05, ** *p* ≤ 0.01, *** *p* ≤ 0.001.

**Table 3:** Correlations among assessments and task performance (*p*-values are shown in parentheses) of both groups.

		Vision	MMSE	Task 1 Efficiency Score	Task 2 Familiar	Task 2 Unfamiliar	Task 3 Face Acc	Task 3 Object Acc
Stroke	Age	−0.603 (0.001) ***	−0.148 (0.294)	0.132 (0.350)	0.325 (0.024) *	0.464 (0.001) **	−0.421 (0.002) **	0.025 (0.859)
	Vision	1	0.216 (0.129)	−0.058 (0.684)	−0.306 (0.037) *	−0.313 (0.027) *	0.262 (0.063)	−0.183 (0.198)
	MMSE	-	1	−0.016 (0.910)	−0.084 (0.570)	−0.042 (0.770)	0.135 (0.338)	0.136 (0.337)
	Efficiency Score	-	-	1	0.052 (0.723)	−0.308 (0.028) *	0.324 (0.019) *	0.136 (0.337)
	Familiar	-	-	-	1	0.517 (0.001) ***	−0.080 (0.589)	0.175 (0.234)
	Unfamiliar	-	-	-	-	1	−0.243 (0.086)	0.088 (0.539)
	Face Acc	-	-	-	-	-	1	0.120 (0.397)
	Object Acc	-	-	-	-	-	-	1
HC	Age	−0.491 (0.033) *	−0.328 (0.088)	−0.438 (0.020) *	−0.025 (0.903)	0.142 (0.473)	−0.433 (0.021) *	0.003 (0.989)
	Vision	1	0.038 (0.879)	0.291 (0.227)	−0.319 (0.198)	−0.244 (0.313)	0.469 (0.043) *	−0.088 (0.719)
	MMSE	-	1	0.168 (0.403)	0.098 (0.633)	−0.264 (0.175)	0.484 (0.009) **	0.364 (0.057)
	Efficiency Score	-	-	1	0.338 (0.092)	0.100 (0.611)	0.104 (0.600)	−0.321 (0.095)
	Familiar	-	-	-	1	0.037 (0.856)	−0.287 (0.155)	−0.134 (0.513)
	Unfamiliar	-	-	-	-	1	−0.467 (0.012) *	−0.165 (0.400)
	Face Acc	-	-	-	-	-	1	−0.272 (0.161)
	Object Acc	-	-	-	-	-	-	1

Task 1: Name that Celebrity (efficiency score); Task 2: Face Identity Card Sorting (number of piles); Task 3: Face/Object Solitaire (accuracy). (* *p* < 0.05, ** *p* ≤ 0.01, *** *p* < 0.001).

## Data Availability

The original datasets (as an Excel file) analyzed during the study have been made publicly available and stored at Open Science Framework via https://osf.io/g95jw/.
